# Fourteen-year trends in overweight, general obesity, and abdominal obesity in Amazonian indigenous peoples

**DOI:** 10.1186/s12889-024-18689-2

**Published:** 2024-05-01

**Authors:** Eliniete de Jesus Fidelis Baniwa, Eliene Rodrigues Putira Sacuena, Rosilene Reis Della Noce, Vanessa Barroso Quaresma, Teodora Honorato Alencar, Renan Barbosa Lemes, Antônia Cherlly Araújo, Izaura Maria Vieira Cayres-Vallinoto, João Farias Guerreiro

**Affiliations:** 1 Laboratory of Human and Medical Genetics, Institute of Biological Sciences, Federal University of Pará, Belém, PA Brazil; 2 Faculty of Nutrition, Institute of Health Sciences, Federal University of Pará, Belém, PA Brazil; 3 Department of Genetics and Evolutionary Biology, Institute of Biosciences, University of São Paulo, São Paulo, SP Brazil; 4 Laboratory of Virology, Institute of Biological Sciences, Federal University of Pará, Belém, PA Brazil; 5 Special Indigenous Health District of Altamira, Special Secretary of Indigenous Health, Altamira, PA Brazil

**Keywords:** BMI, Overweight, Obesity, Indigenous people, Brazilian Amazon

## Abstract

**Background:**

Available data show that the epidemiological profile of most indigenous Brazilian populations is characterized by the coexistence of long-standing health problems (high prevalence of infectious and parasitic diseases, malnutrition, and deficiency diseases, such as anemia in children and women of reproductive age), associated with new health problems, especially those related to obesity (hypertension, type 2 diabetes mellitus and dyslipidemia). Based on this scenario, this study analyzed the nutritional profile of the adult population of seven indigenous peoples from the Brazilian Amazon in the years 2007 and 2021.

**Methods:**

A total of 598 adults individuals were analyzed in 2007 (319 women and 279 men) and 924 in 2021 (483 women and 441 men), from seven indigenous peoples located in the state of Pará, who were assisted during health actions carried out in 2007 and in 2021. Body mass index classification used the World Health Organization criteria for adults: low weight, < 18.5 kg/m^2^; normal weight, ≥ 18.5 and < 25 kg/m^2^); overweight, ≥ 25 and < 30 kg/m^2^, and obesity, ≥ 30 kg/m^2^. A waist circumference (WC) < 90 cm in men and < 80 cm in women was considered normal.

**Results:**

The data revealed heterogeneous anthropometric profiles, with a low prevalence of nutritional changes in the Araweté, Arara and Parakanã peoples, and high proportions of excess weight and abdominal obesity in the Kararaô, Xikrin do Bacajá, Asurini do Xingu and Gavião peoples, similar to or even higher than the national averages.

**Conclusion:**

Different stages of nutritional transition were identified in the indigenous peoples analyzed, despite apparently having been subjected to the same environmental pressures that shaped their nutritional profile in recent decades, which may indicate different genetic susceptibilities to nutritional changes. The evidence shown in this study strongly suggests the need to investigate in greater depth the genetic and environmental factors associated with the nutritional profile of Brazilian indigenous peoples, with assessment of diet, physical activity and sociodemographic and socioeconomic variables that enable the development of appropriate prevention and monitoring measures.

**Supplementary Information:**

The online version contains supplementary material available at 10.1186/s12889-024-18689-2.

## Introduction

The epidemiological scenario in the Brazilian population in general is characterized by a transition in the pattern of mortality and morbidity from infectious and parasitic diseases (DIP) to a profile with a predominance of c (NCDs) such as general obesity, type 2 diabetes mellitus (T2DM), systemic arterial hypertension (SAH) and cancer, although there are different transition patterns in different areas of the country due to differences in the level of regional and social development [1].

Among indigenous peoples, on the other hand, infectious and parasitic diseases are still the main causes of morbidity and mortality, but there is evidence of an epidemiological transition with an increase in the prevalence of NCDs, in addition to the presence of mental and behavioral disorders and so-called social pathologies – violence and negative effects of alcohol and drug abuse.

Chronic noncommunicable diseases began to be observed in Brazilian indigenous populations only at the end of the 1970s. In the Brazilian Amazon, the first reference to diabetes was made in 1977 in the Karipúna and Palikúr peoples, in the state of Amapá [1]. The first description of arterial hypertension was made among the Terena, in the state of Mato Grosso [2]. Cases of obesity began to be reported at the end of the 1990s in Suruí, the state of Rondônia [3] and in Tembé, the state of Pará [4]. Since the 2000s, the presence of such chronic diseases has been described in an increasing number of indigenous groups in varying proportions [5–7]. Available data reveal a dramatic increase in the rate of chronic noncommunicable diseases following the rapid nutritional transition to more urban diets among Brazilian indigenous peoples, with a direct association between urbanization and the consequent macrosocial changes in the traditional way of life of these people, with the increased prevalence of cardiometabolic risks among them. In this context, urbanization refers both to the adoption of urban lifestyles in villages and to housing in urban regions of Brazil ((i.e., where city dwellers live). This implies living conditions integrated with the market and less dependence on local food production, reducing food sovereignty, which involves rights to food autonomy and healthy diets, especially among women, for whom the available data point to distinct patterns of associations between socioeconomic indicators and the occurrence of overweight and obesity, which have potentially significant implications from the point of view of public policies for indigenous peoples in Brazil [6, 8, 9]. Furthermore, there is evidence that social changes due to contact with urban lifestyles, historical conflicts, loss of territory, and cultural disruption, observed in several indigenous peoples, are implicated in the increase in alcohol consumption, resulting in interpersonal violence, in disruptions in family life and in accidents, which have become part of the daily lives of some indigenous peoples [10]. There is also evidence that changes in the lifestyle of indigenous people have led to the introduction of foods that are ultra-processed, high calorie foods, and/or high in refined carbohydrates, sugar, and saturated fat; evidence also suggests that there has been a reduction in the frequency and intensity of physical activities in the face of technological advances, such as the use of automobiles and outboard engines for transportation [2–4]. Therefore, significant changes are observed in the epidemiological profile of Brazilian indigenous peoples, mainly with the increase in the prevalence of general obesity and comorbidities, changes that are comparable to those that affected native North Americans in the second half of the twentieth century, during which important sociocultural changes have led to a dramatic increase in chronic diseases [5]. Similarly, in Latin America, other indigenous populations have also undergone similar changes and currently exhibit a high prevalence of general obesity and, in some cases, T2DM [6].

Based on this complex scenario, this study was carried out with the objective of investigating the nutritional profile, by measuring the body mass index and waist circumference, of the adult population of seven indigenous peoples in the state of Pará, in the Brazilian Amazon in the years 2007 and 2021, and identifying trends that may justify in-depth evaluation of the biological and environmental factors, as well as the social determinants associated with the nutritional profile of these people. This study aims to enable the organization of prevention programs that are more appropriate to containing or minimizing this epidemiological trend.

## Material and methods

### Study population

A total of 598 indigenous adults were analyzed in 2007 (319 women and 279 men) and 924 in 2021 (483 women and 441 men), who belonged to seven peoples located in the State of Pará (PA), in the Brazilian Amazon:

- Arara: Karib-speaking, with a total population of 349 individuals in 2021.They currently live in six villages in the Arara Indigenous Territory, on the banks of the Iriri River, a tributary of the Xingu River, in the municipality of Altamira.
- Araweté: They speak a language in the Tupi-Guarani family and had a total population of 559 people in 2021. The Araweté live in twelve villages in the Araweté/Igarapé Ipixuna Indigenous Territory, on the banks of the Xingu River and Igarapé Ipixuna, a tributary on the right bank of the Middle Xingu, municipality of Altamira.
- Asurini do Xingu: They speak a language in the Tupi-Guarani family and had a total population of 260 people in 2021, Live in the Koatinemo Indigenous Territory, located on the banks of the Xingu River, close to Igarapé Ipixuna, municipality of Altamira.
- Parakanã: They speak a language in the Tupi-Guarani family and had a total population of 716 people in 2021., Currently living in 11 villages in the Apyterewa Indigenous Territory, located in the Xingu basin, in the municipality of São Félix do Xingu.
- Kararaô: They speak a language in the Jê family. This small Kayapó subgroup (Mebêngôkre Kayapó Kararaô), with a total population of eighty Indigenous people in 2021, was created by from a split of the Gorotire group that occurred at the beginning of the twentieth century. They live in three villages located in the Kararaô Indigenous Territory, on the banks of the Iriri and Xingu Rivers.
- Xikrin do Bacajá: They speak the Kayapó language, from the Jê linguistic family, and they had a total population of 1127 in the 2021. This Kayapó subgroup is distributed in eleven villages on the banks of the middle Bacajá River, a tributary of the right bank of the Xingu River, in the Trincheira Bacajá Indigenous Territory, which is in the municipalities of Senator José Porfírio and Anapú.
- Gavião: They speak East Timbira, from the Jê family, and they had a total population of 760 in 2021. They live in the Mãe Maria Indigenous Territory, which is located on the border of the municipalities of Marabá and Bom Jesus do Tocantins, southeast of Pará.

**Fig. 1:**
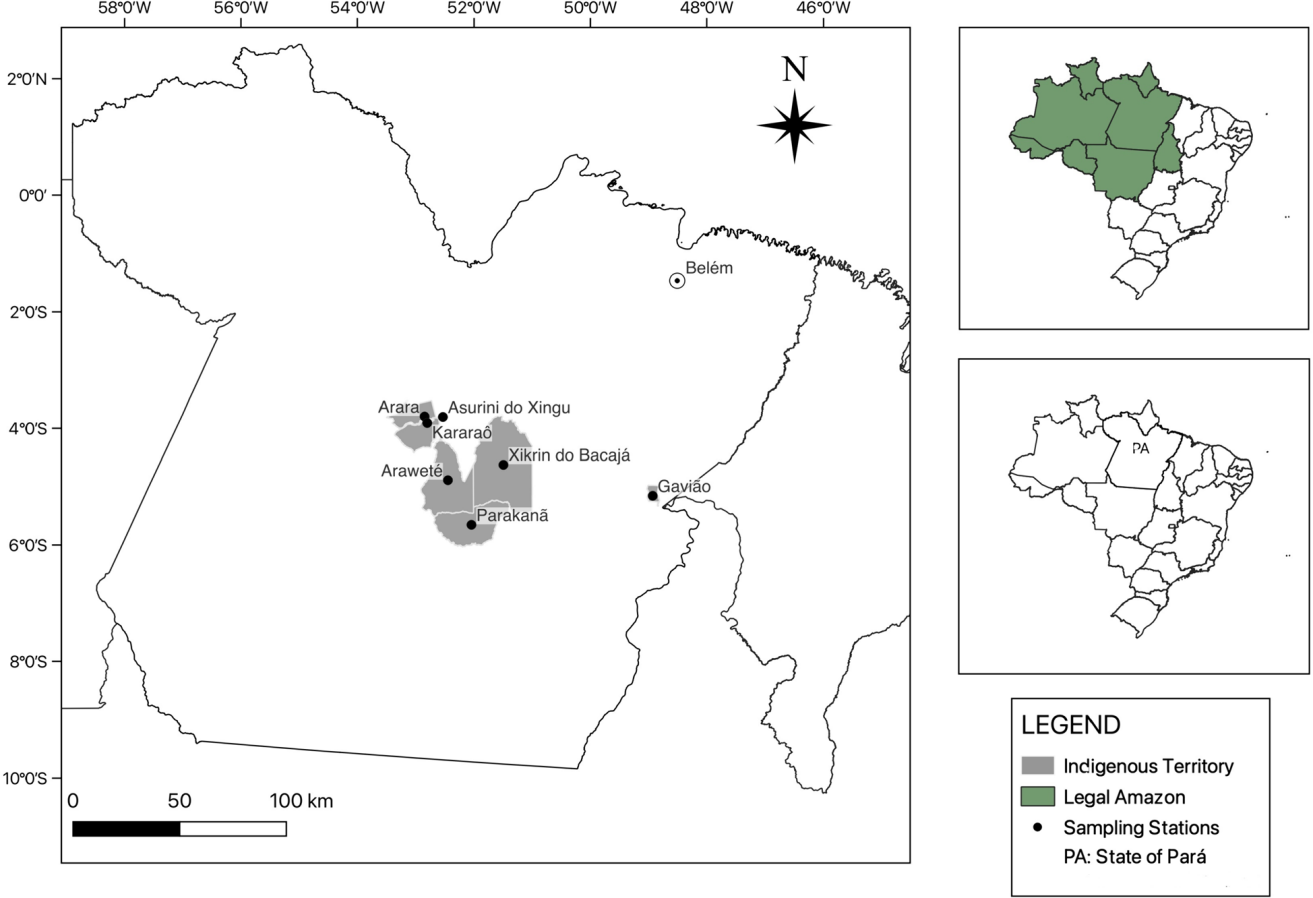
Geographical location of the studied indigenous peoples in the state of Pará, in the Brazilian Amazon

The geographic locations of the indigenous territories analyzed are shown in Fig. 1.

Participants were selected based on spontaneous demand during basic health actions and epidemiological studies conducted with indigenous peoples in cooperation with the National Health Foundation (FUNASA), in 2007, and with the State Secretariat of Health of Pará (SESPA) and Special Indigenous Health Districts (DSEI/SESAI) in 2021, during which demographic and clinical-epidemiological data were obtained. During fieldwork, all people aged six months or older or their guardians were invited to perform an anthropometric assessment and blood (5 mL) were collected in vacuum collection tubes; for children drops of blood were obtained by digital puncture for examination. Hematological and/or biochemical tests were conducted, and the patients were duly instructed on the need to fast before conducting biochemical tests. The adherence of indigenous communities to health actions was high and the percentage of participants among adults eligible for nutritional and biochemical assessment in all populations was approximately 70%. All participants were evaluated in a medical consultation, and treatment began for conditions that could be confirmed in the field.

Body mass index (BMI) classification was based on the World Health Organization (WHO) criteria for adults: low weight, < 18.5 kg/m^2^; normal weight, ≥ 18.5 and < 25 kg/m^2^); overweight, ≥ 25 and < 30 kg/m^2^, and obesity, ≥ 30 kg/m^2^. A waist circumference (WC) < 90 cm in men and < 80 cm in women was considered normal. Pregnant women were not included in the sample analyzed and no specific BMI classification for the elderly population was used.

Weight was measured with the individual barefoot, standing upright, in the center of the equipment, with feet together and arms extended along the body. Height was measured using a stadiometer in centimeters with the patient standing upright, with arms extended along the body, head raised, and looking at a fixed point at eye level, without flexing or extending the head. WC was obtained using inelastic tape, with the patient standing, with the tape surrounding the waist at the midpoint between the last rib and the iliac crest.

### Statistical analysis

Biological data were compared using Student’s t test for continuous variables and Pearson’s χ2 test, Fisher’s exact test or ANOVA for categorical variables. An overall significance level of 0.05 was established for statistical analyses. The data was analyzed using IBM SPSS Statistics for Windows (Version 20).

## Results

### Anthropometric variables

Anthropometric data were obtained from 598 Indigenous adults in 2007 (319 women and 279 men), aged between 18 and 88 years, with a mean of 38.8 years (95% CI 57.3, 74.7), and from 881 in 2021 (463 women and 420 men), aged between 18 and 102 years, with average of 37.7 years (95% CI, 36.5–38.8).

The average values of the anthropometric variables are presented in Tables 1 and S1.

**Table 1:** Mean values of weight, height, body mass index (BMI) and waist circumference (WC) in seven indigenous people from the Brazilian Amazon in 2007 and 2021

People	Year	Weight (kg)			Height (m)			BMI (kg/m2)			WC (cm)		
		N	Mean	CI 95%	N	Mean	CI 95%	N	Mean	CI 95%	N	Mean	CI 95%
Arara	2007	65	54.6	38.4–70.7	65	1.55	1.4–1.7	65	22.7	17.5–28.0	44	80.4	69.3–91.6
	2021	82	57.4	41.6–73.2	82	1.53	1.4–1.7	82	24.4	18.3–30.5			-
Araweté	2007	65	51.3	38.1–64.5	65	1.54	1.4–1.7	65	21.7	18.0–25.5	64	78.6	67.1–90.1
	2021	217	52.5	33.4–71.6	216	1.53	1.4–1.7	214	22.3	15.3–29.2	215	78.4	61.0–95.9
Asurini	2007	37	62.2	33.9–90.6	37	1.54	1.4–1.7	37	26.1	16.0–36.1	37	91.1	70.0–112.1
	2021	58	69.7	43.1–96.4	57	1.62	1.5–1.8	58	26.4	19.4–33.5	58	92.1	64.6–119.7
Kararaô	2007	11	68.7	46.3–91.0	11	1.59	1.5–1.7	11	27.1	18.7–35.5	11	87.8	68.2–107.5
	2021	23	73.8	40.9–106.7	23	1.58	1.4–1.7	23	29.1	17.7–40.6	23	92.5	67.1–118.0
Xikrin	2007	159	62.8	43.4–82.2	158	1.60	1.5–1.7	157	24.4	17.9–30.9	157	83.7	59.2–108.2
	2021	165	69.0	42.0–96.0	164	1.58	1.4–1.7	164	27.6	17.4–37.9	157	90.8	57.6–124.0
Parakanã	2007	123	56.2	39.5–72.9	123	1.54	1.4–1.7	123	23.6	17.7–29.4	127	81.3	64.8–97.9
	2021	142	62.8	33.3–92.2	91	1.54	1.4–1.7	85	25.8	16.9–34.7	73	75.2	44.0–106.3
Gavião	2007	61	68.1	38.8–97.3	59	1.62	1.4–1.8	59	26.6	17.2–35.9	50	93.7	72.0–115.4
	2021	136	74.6	43.7–105.5	127	1.62	1.4–1.8	124	29.0	19.9–38.1	108	81.5	44.0–119.1

In 2007, the lowest average body weights were recorded for the Araweté (51.3 kg, 95% CI 38.1–64.5), Arara (54.6 kg, 95% CI 38.4–70.7), and Parakanã (56.2 kg, 95% CI 39.5–72.9) peoples, and the highest were found for the Gavião (68.1 kg, 95% CI 38.8–97.3) and Kararaô (68.7, 95% CI 46.3–91.0) peoples.

The lowest mean BMI values were also found for the Araweté (21.7 kg/m^2^, 95% CI 18.0–25.5) and Arara (22.7 kg/m^2^, 95% CI 17.5–28.0) peoples. Slightly higher values were found for the Parakanã (23.6 kg/m^2^, 95% CI 17.7–29.4) and Xikrin do Bacajá (24.4 kg/m^2^, 95% CI 17.9–30.9) peoples, while values above 25 kg/m^2^, the cutoff point for overweight, were recorded among the Asurini do Xingu, Kararaô and Gavião peoples. The Araweté, Arara and Parakanã peoples also had the lowest mean WC values (78.6 cm, 95% CI 67.1–90.1; 80.4 cm, 95% CI 69.3–91.6; and 81.3 cm, 95% CI, 64.8–97.9, respectively). A slightly greater value was recorded for the Xikrin do Bacajá (83.7 cm, 95% CI 59.2–108.2), while Asurini do Xingu and Gavião peoples had higher average values, above 90 cm (91.1 cm, 95% CI 70.0–112.1, and 93.7 cm, 95% CI 72.0–115.4, respectively).

From 2007 to 2021, the average body weight increased in all indigenous peoples analyzed. The Araweté and Arara peoples continued to exhibit lower average weights (52.5 kg, 95% CI 33.4–71.6, and 57.4 kg, 95% CI 41.6–73.2, respectively), and Gavião and Kararaô peoples had the highest average weights (74.6 kg, 95% CI 43.7- 105.5, and 73.8 kg, 95% CI 40.9–106.7, respectively).

The lowest mean BMI values also continued to be found in the Araweté (22.3 kg/m^2^, 95% CI 15.3–29.2) and Arara (24.4 kg/m^2^ CI95% 18.3–30.5) peoples, while mean values above 25 kg/m^2^, the threshold for overweight, were observed in all other indigenous peoples, with values varying from 25.8 kg/m^2^, 95% CI 16.9–34.7 in the Parakanã people, to 29.1 kg/m^2^ 95% CI 17.7–40.6 in the Kararaô people. The average WC did not change in Araweté and Asurini do Xingu peoples, from 2007 to 2021, it increased in Kararaô and Xikrin do Bacajá, but decreased in the Parakanã and Gavião peoples.

### Nutritional status indicators

**Fig. 2:**
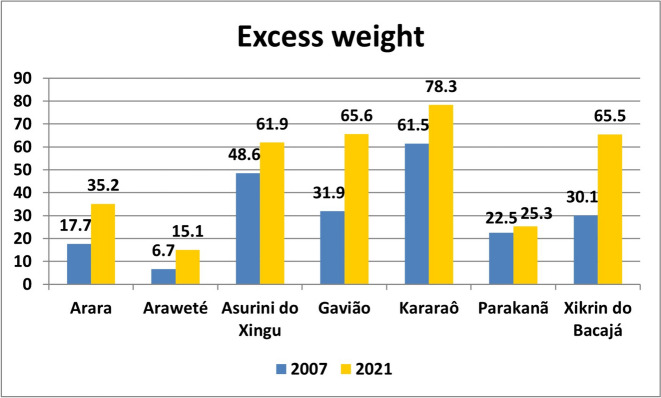
Prevalence of excess weight in Amazonian indigenous peoples in 2007 and 2021

**Fig. 3:**
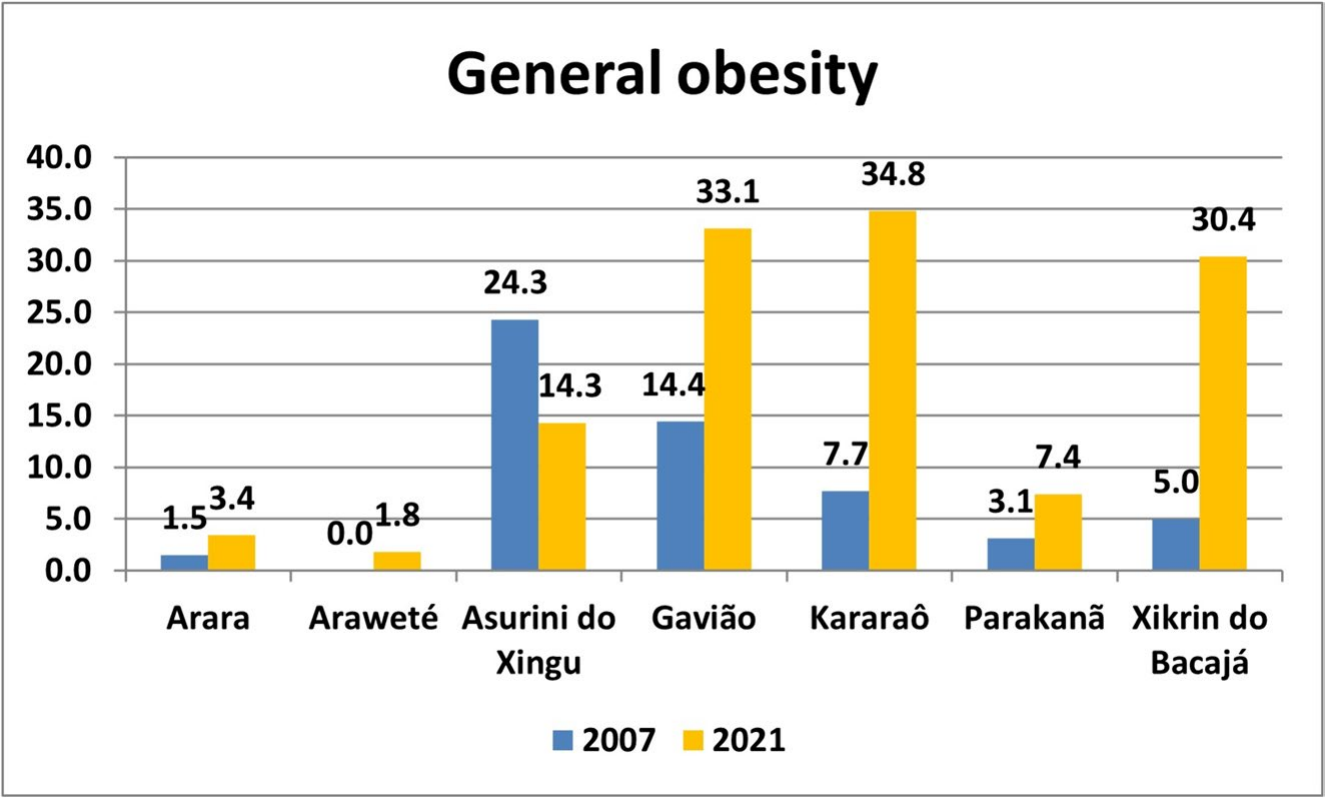
Prevalence of general obesity in Amazonian indigenous peoples in 2007 and 2021

**Fig. 4:**
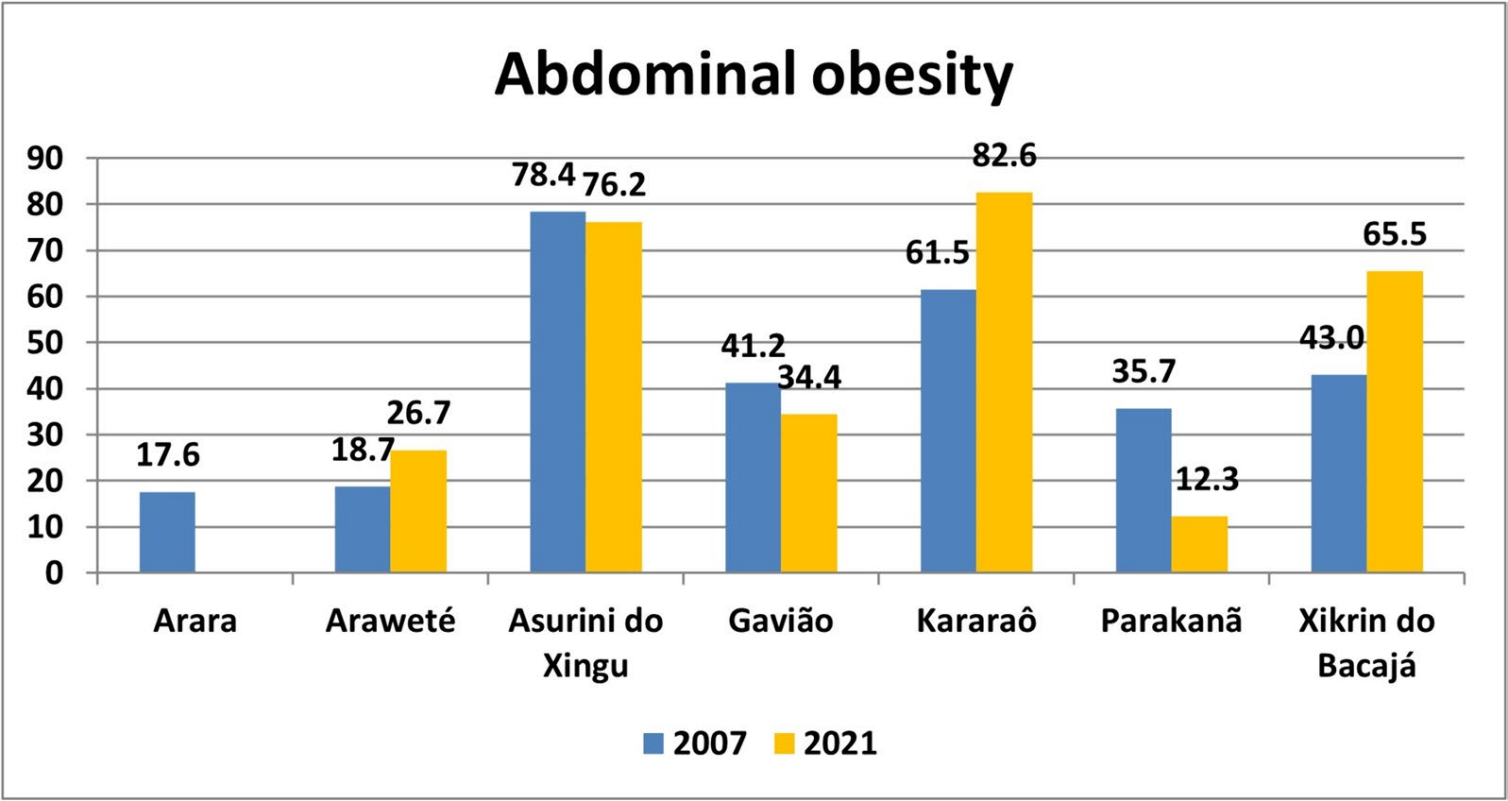
Prevalence of abdominal obesity in Amazonian indigenous peoples in 2007 and 2021

The prevalence of excess weight, general obesity and abdominal obesity are presented in Figs. 2, 3 and 4 and Table S2.

In 2007, the prevalence of excess weight (overweight and obesity) was low in most of the Indigenous peoples analyzed. The lowest prevalence was observed in the Araweté people (6.7%), and moderate prevalences were found in the Arara (17.7%), Parakanã (22.5%), Xikrin do Bacajá (30.1%) and Gavião (31.9%) peoples. High proportions were found in the Asurini do Xingu (48.6%) and the Kararaô (61.5%) peoples. Increases in the prevalence of excess weight from 2007 to 2021 occurred in all Indigenous peoples analyzed, but values remained low or moderate in the Araweté (15.1%), Parakanã (25.3%) and Arara (35.2%) peoples. Among the other indigenous peoples analyzed, the increases were more significant, reaching prevalences above 60%, in particular the increase observed among the Xikrin do Bacajá people, which ranged from 30.1% to 65.5%. The data analysis did not show significant differences in the prevalence of excess weight between men and women in any of the indigenous peoples analyzed in 2007 (*p* > 0.05). In 2021, a significant difference was observed only among the Asurini do Xingu people, with the prevalence of excess weight being greater among men (68.4% versus 55.2%, *p* = 0.045) (Fig. 2).

The prevalence of general obesity in 2007 was also low in most of the indigenous peoples analyzed, with the highest being found in the Gavião (14.4%) and the Asurini do Xingu (24.3%) peoples. From 2007 to 2021, increases were recorded in almost all populations, but the most significant increase occurred in the Gavião (from 14.4% to 33.1%), Kararaô (from 7.7% to 34.8%), and Xikrin of Bacajá (from 5.0% to 30.4%) peoples. There were also no significant differences in the prevalence of general obesity between men and women in any of the indigenous peoples analyzed in 2007 (*p* > 0.05). However, in 2021, a significant difference was observed in the Xikrin do Bacajá people, with a greater prevalence of general obesity in women (37.8% versus 23.0%, *p* = 0.044) (Fig. 3).

In 2007, the highest prevalence rates of abdominal obesity were found in the Asurini doXingu (78.4%) and Kararaô (61.5%) peoples, and the lowest were found in the Arara (17.6%) and Araweté (18.7%) peoples. In 2021, significant increases in the prevalence of abdominal obesity were observed in the Xikrin do Bacajá (from 43.0% to 65.5%) and Kararaô (from 61.5% to 82.6%) peoples, which together with the Asurini do Xingu people (76.2%) exhibited the highest prevalence among the people analyzed. There was a slight decrease in the prevalence of abdominal obesity among the Gavião people (from 41.4% to 34.4%), but attention was given to the significant decrease in the prevalence of this condition among the Parakanã people, which decreased from 35.7 to 12.3%, the lowest prevalence of abdominal obesity among the seven indigenous peoples analyzed. Waist circumference was not measured in Arara people in 2021. Contrary to what was observed for BMI, the prevalence of abdominal obesity was significantly greater in women than in men in almost all indigenous peoples, both in 2007 and 2021, except for the Kararaô people, in which the prevalence among men and women were similar, with *p* = 0.491 in 2007 and *p* = 1.000 in 2021 (Fig. 4).

## Discussion

The epidemiological data obtained in this study showed that despite the significant increase in the occurrence of excess weight (overweight/obesity) in the majority of indigenous peoples analyzed from 2007 to 2021, relatively low prevalences were still found in 2021 among peoples such as the Araweté, Arara and Parakanã peoples, as well as high prevalences, which were higher than the average recorded for Brazilian urban adults (52.5%) [9] in the Xikrin do Bacajá, Asurini do Xingu, Kararaô and Gavião peoples. The prevalence of general obesity in the Araweté, Arara and Parakanã peoples in 2021 was also low; however, the prevalence rates in three of the seven populations analyzed (the Gavião, Kararaô and Xikrin do Bacajá poples), were higher than the average of 23.9% for the Brazilian adult population in general [9]. High prevalences of abdominal obesity, which were equal to or higher than the national average of 69.2% [10] were also recorded in 2021 among the Xikrin do Bacajá, Kararaô and Asurini do Xingu peoples; however, again, low prevalences were identified among the Araweté, Arara and Parakanã peoples.

The data obtained suggest that although the indigenous peoples analyzed were subjected to environmental conditions and sociocultural changes that favored weight gain between 2007 and 2021, which impacted nutritional assessment measures, the nutritional profiles of the indigenous peoples analyzed was heterogeneous. People such as the Araweté, Arara and Parakanã still had a low prevalence of overweight and abdominal obesity in 2021, and people such as the Gavião, Kararaô, Asurini do Xingu and Xikrin do Bacajá exhibited a high prevalence of nutritional changes, similar to or greater than those registered for the Brazilian population in general.

These data suggest that genetic factors play a significant role in the development of nutritional changes among indigenous peoples, modulating responses to environmental pressures, which were apparently the same in all indigenous peoples analyzed. An interesting aspect of this scenario is that the people who exhibited a low or moderate prevalence of nutritional changes are speakers of the Karib language, such as the Arara people, or speakers of the Tupi-Guarani family, such as the Araweté, Parakanã and Asurini do Xingu peoples. On the other hand, people such as Gavião, Kararaô and Xikrin do Bacajá, who exhibited the highest prevalence of overweight and general obesity in 2021, are speakers of language of the Jê family, similar to the Xavante people, from Mato Grosso, who also exhibit high prevalence of nutritional changes [4].

It is widely known that environmental factors contribute to weight gain, including a sedentary lifestyle, eating high-calorie/nutrient-poor foods, and reduced energy expenditure, but it is also well-known that genetics contribute to determining an individual’s response to an ‘obesogenic environment’. In global terms, it is assumed that genetic components characterize 30–55% of the body fat distribution. Obesity is usually classified into two broad categories: monogenic and polygenic. The monogenic form is inherited by Mendelian inheritance, that is, it is caused by a mutation in a certain gene. These are rare forms and present at a relatively severe and early age of onset. The most common form of obesity is polygenic, driven by hundreds or possibly thousands of mutations known as single nucleotide polymorphisms (SNPs), each with a small additive effect and distributed throughout the human genome. Therefore, it has a complex mode of inheritance typical of common characteristics. Currently it is known that another type of gene-environment interaction (GEI) that leads to obesity and various metabolic disorders, known as epigenetic interaction, produces reversible changes in gene activity, but without altering the DNA sequence; these genes changes are hereditary, using mechanisms such as DNA methylation and hydroxymethylation, gene regulation of noncoding RNAs and modification of chromatin and histones). From a population perspective, epidemiological data reveal that certain ethnic groups are more (or less) likely to become obese, and these data indicate a greater prevalence of obesity in Native Americans, as well as in “Latinos” or “Mestizos” (resulting from the mixture between Native Americans and Europeans), than in Euro-Americans, and data reveal that the risk for these pathologies increases with a increase in indigenous ancestry. Despite this, the available genetic data result from studies carried out predominantly in European populations, and in Asians to a lesser extent, while Latin populations and Native American populations have been less studied [11].

Although the available data on nutritional anthropometry in Brazilian indigenous peoples is limited and poorly updated, it is possible to compare the results obtained in this study in 2021 with some slightly more recent data recorded for other Indigenous peoples.

The low prevalence of excess weight (overweight/obesity) found in the Araweté does not have comparable results among those described more recently in other Indigenous peoples, possibly constituting a rare finding in indigenous people who already maintain open contact with the national society. The prevalence found in the Arara people is also still lower than that described in recent years for Brazilian indigenous peoples, which ranges from 47.3% in Suyá-Khisêdjê people, Mato Grosso [12], to 85.5% in the Xavante, also from Mato Grosso [13]. On the other hand, the high prevalence of excess weight observed in the Gavião, Kararaô, Asurini do Xingu and Xikrin do Bacajá peoples are similar to those recorded in peoples such as the Xavante, Mato Grosso (85.5%) [13], Xikrin do Cateté, Pará (78.0%) [14], Kaingang, Santa Catarina (67.1%) [15], Kaiowá, Guarani, and Terena, Mato Grosso (61.3%) [16], in which the prevalence of excess weight are higher than the global average of 52.5% estimated for the Brazilian population [17].

The prevalences of general obesity recorded in the Araweté and Arara peoples in 2021 are also lower than those found in most indigenous peoples for whom recent data are available and are only similar to that recorded for Suyá-Khisêdjê people, Mato Grosso (5.3%) [12]. On the other hand, the high proportions of general obesity observed in 2021 for the Kararaô, Asurini do Xingu and Gavião peoples, are similar to those found in other indigenous peoples such as the Xikrin do Cateté (36.0%), Pará [14], Kaiowá, Guarani and Terena (37.0%), from Mato Grosso [15], Kaingang (33.1%), from Santa Catarina [15] and Xavante (51%), from Mato Grosso [13].

The relatively low proportions of abdominal obesity identified in the Araweté and Parakanã peoples in 2021 are also similar to those found in the Suyá-Khisêdjê people (13.3%), from Mato Grosso [12]. On the other hand, the proportions of WC found among the Asurini do Xingu, Kararaô and Xikrin do Bacajá peoples are greater than those found in other indigenous peoples such as the Kaiowa, Guarani and Terena (56.0%), Mato Grosso [16] and Mura (48.6%), Amazonas [18], and are more similar to those described for the Xikrin do Cateté (80.7%), Pará [14] and Xavante, Mato Grosso (96.2%); these are the people who exhibit the highest prevalence of abdominal obesity among indigenous Brazilians, which are similar or even greater than the average for the general Brazilian population (80.7%).

In the context of social determinants of health, it is important to consider that the indigenous peoples covered in this study have historically had distinct levels of interethnic relations, but in recent decades they have shared experiences of environmental and/or sociocultural impacts caused mainly by large economic projects. Furthermore, the people who belong to the “Médio Xingu” ethnographic area (Asurini do Xingu, Araweté, Parakanã, Xikrin do Bacajá, Kararaô and Arara) live in indigenous territories that are located in an area characterized by intense logging, mining, and agricultural activity, in addition to having been impacted socioculturally and economically by the remuneration policies used by the company “Norte Energia” for the construction of the Belo Monte Hydroelectric Plant; these groups have been even more affected since 2016, with the allocation of financial resources and donation of food containing ultra-processed items. The Gavião people, in turn, were impacted environmentally and socioculturally due to the construction of the BR-322 highway, the installation of electrical power transmission lines and the construction of a railway to transport iron to serve the Carajás Project, which is a large mineral extraction project that started in the in 1980s, and that caused not only environmental degradation, but also cultural and identity-related impacts Additionally, there are effects that have been generated by financial resources arising from compensation negotiated by the indigenous people with Eletronorte Company, a public electricity service concessionaire, and the “Vale do Rio Doce” Company, which is responsible for the Carajás Project. Regardless of the large economic project installed in the Indigenous Territories, one of the most visible effects, which certainly contributed to the change in the nutritional profile of most peoples, was the large contribution of financial resources to the indigenous people, generating a growing demand for money and goods, such as industrialized foods, including high-calorie foods such as soft drinks and cookies.

This study has some limitations. The most important is that the samples studied in 2007 and 2021 were not homogeneous in some indigenous peoples in terms of sex ratio. However, this is a complicated issuesince indigenous people are always dividing and forming new villages, and it is not always possible to access all of them. Despite this, the epidemiological data obtained in this study show that the nutritional profile of the investigated indigenous populations s heterogeneous; the data show populations with a low prevalence of nutritional changes, such as the Araweté,Arara, and Parakanã peoples, while other such as the Kararaô, Xikrin do Bacajá, Asurini do Xingu, and Gavião peoples, already exhibited high frequencies of excess weight and abdominal obesity, even though these people apparently had already been subjected to the same environmental pressures that have promoted changes in their lifestyles in recent decades. In this context, the evidence presented in this study strongly suggests the need to understand in greater depth the genetic and environmental factors and social determinants associated with the nutritional profile of these people, with a better assessment of diet and physical activities, in particular, which would make it possible to organize adequate prevention programs to meet the needs of these people.

### Supplementary Information

**Supplementary Material 1.**

**Supplementary Material 2.**

## Data Availability

All data generated or analyzed during this study are included in this published article and its supplementary information files.
