# Supplementary material for: Fourteen-year trends in overweight, general obesity, and abdominal obesity in Amazonian indigenous peoples
Source: BMC Public Health. 2024 May 1;24:1210. doi: 10.1186/s12889-024-18689-2 (PMC11064236; doi:10.1186/s12889-024-18689-2)
Supplement: Supplementary file 1 — Supplementary Material 1. [file 12889_2024_18689_MOESM1_ESM.docx]

Table S1. Mean values of age, weight, height, and BMI, by sex, in seven indigenous people from the Brazilian Amazon in 2007 and 2021.

| People | Year | Sex | Age (Years) | | | Weight (kg) | | | Height (m) | | | BMI (kg/m2) | | |
| --- | --- | --- | --- | --- | --- | --- | --- | --- | --- | --- | --- | --- | --- | --- |
|  |  |  | N | Mean | CI 95% | N | Mean | CI 95% | N | Mean | CI 95% | N | Mean | CI 95% |
| Arara | 2007 | Female | 36 | 40.4 | 9.0 71.7 | 33 | 49.5 | 38.0-61.0 | 33 | 1.5 | 1.4-1.6 | 33 | 22.8 | 17.6-28.1 |
|  | 2007 | Male | 32 | 39.6 | 4.6 74.5 | 32 | 59.8 | 46.1-73.5 | 32 | 1.6 | 1.5-1.7 | 32 | 22.6 | 17.3-27.9 |
|  | 2007 | Total | 68 | 40.0 | 7.2 72.8 | 65 | 54.6 | 38.4-70.7 | 65 | 1.6 | 1.4-1.7 | 65 | 22.7 | 17.5-28.0 |
|  | 2021 | Female | 48 | 36.7 | 5.6 67.8 | 45 | 54.6 | 40.9-68.4 | 45 | 1.5 | 1.4-1.6 | 45 | 24.6 | 18.1-31.0 |
|  | 2021 | Male | 40 | 35.8 | -6.9 78.5 | 37 | 60.8 | 45.0-76.5 | 37 | 1.6 | 1.5-1.7 | 37 | 24.2 | 18.6-29.8 |
|  | 2021 | Total | 88 | 36.3 | -0.3 72.9 | 82 | 57.4 | 41.6-73.2 | 82 | 1.5 | 1.4-1.7 | 82 | 24.4 | 18.3-30.5 |
| Araweté | 2007 | Female | 35 | 43.1 | 11.9 74.3 | 30 | 47.2 | 35.5-59.0 | 30 | 1.5 | 1.4-1.6 | 30 | 21.9 | 17.2-26.5 |
|  | 2007 | Male | 40 | 49.7 | 14.4 85.0 | 35 | 54.8 | 44.4-65.1 | 35 | 1.6 | 1.5-1.7 | 35 | 21.6 | 18.7-24.4 |
|  | 2007 | Total | 75 | 46.6 | 12.8 80.5 | 65 | 51.3 | 38.1-64.5 | 65 | 1.5 | 1.4-1.7 | 65 | 21.7 | 18.0-25.5 |
|  | 2021 | Female | 120 | 37.3 | 5.1 69.5 | 115 | 49.9 | 32.0-67.8 | 115 | 1.5 | 1.4-1.6 | 113 | 22.4 | 15.7-29.2 |
|  | 2021 | Male | 105 | 38 | 1.5 74.5 | 102 | 55.5 | 36.7-74.3 | 101 | 1.6 | 1.5-1.7 | 101 | 22.1 | 14.8-29.3 |
|  | 2021 | Total | 225 | 37.6 | 3.4 71.8 | 217 | 52.5 | 33.4-71.6 | 216 | 1.5 | 1.4-1.7 | 214 | 22.3 | 15.3-29.2 |
| Asurini | 2007 | Female | 22 | 48.2 | 17.1 79.3 | 22 | 58.9 | 30.9-86.8 | 22 | 1.5 | 1.4-1.6 | 22 | 26.3 | 15.2-37.3 |
|  | 2007 | Male | 15 | 46.1 | 8.8 83.5 | 15 | 67.2 | 40.3-94.1 | 15 | 1.6 | 1.5-1.7 | 15 | 25.8 | 17.2-34.4 |
|  | 2007 | Total | 37 | 47.4 | 14.0 80.7 | 37 | 62.2 | 33.9-90.6 | 37 | 1.5 | 1.4-1.7 | 37 | 26.1 | 16.0-36.1 |
|  | 2021 | Female | 54 | 38.2 | 1.4 75.1 | 29 | 63 | 42.2-83.8 | 29 | 1.6 | 1.4-1.7 | 29 | 25.6 | 19.0-32.2 |
|  | 2021 | Male | 52 | 33.3 | 4.6 61.9 | 29 | 76.5 | 50.9-102.0 | 28 | 1.7 | 1.5-1.8 | 29 | 27.3 | 20.1-34.4 |
|  | 2021 | Total | 106 | 35.8 | 2.5 69.1 | 58 | 69.7 | 43.1-96.4 | 57 | 1.6 | 1.5-1.8 | 58 | 26.4 | 19.4-33.5 |
| Kararaô | 2007 | Female | 8 | 41.4 | 5.5 77.2 | 7 | 67.2 | 44.8-89.5 | 7 | 1.6 | 1.4-1.7 | 7 | 27.4 | 17.5-37.3 |
|  | 2007 | Male | 5 | 43.2 | -6.3 92.7 | 4 | 71.3 | 46.6-96.0 | 4 | 1.6 | 1.5-1.8 | 4 | 26.5 | 20.4-32.6 |
|  | 2007 | Total | 13 | 42.1 | 2.5 81.7 | 11 | 68.7 | 46.3-91.0 | 11 | 1.6 | 1.5-1.7 | 11 | 27.1 | 18.7-35.5 |
|  | 2021 | Female | 16 | 38.8 | -0.3 77.9 | 16 | 71.4 | 38.5-104.2 | 16 | 1.6 | 1.4-1.7 | 16 | 28.9 | 17.9-39.9 |
|  | 2021 | Male | 7 | 36.9 | 15.2 58.5 | 7 | 79.3 | 46.6-112.0 | 7 | 1.6 | 1.5-1.8 | 7 | 29.7 | 16.5-42.9 |
|  | 2021 | Total | 23 | 38.2 | 4.0 72.5 | 23 | 73.8 | 40.9-106.7 | 23 | 1.6 | 1.4-1.7 | 23 | 29.1 | 17.7-40.6 |
| Xikrin | 2007 | Female | 92 | 37.2 | 4.8 69.6 | 81 | 58.5 | 39.2-77.9 | 80 | 1.5 | 1.5-1.6 | 80 | 24.3 | 16.9-31.7 |
|  | 2007 | Male | 87 | 35.3 | 3.8 66.8 | 78 | 67.1 | 51.7-82.5 | 78 | 1.7 | 1.6-1.8 | 77 | 24.5 | 19.0-30.0 |
|  | 2007 | Total | 179 | 36.3 | 4.4 68.2 | 159 | 62.8 | 43.4-82.2 | 158 | 1.6 | 1.5-1.7 | 157 | 24.4 | 17.9-30.9 |
|  | 2021 | Female | 92 | 34.6 | 3.4 65.9 | 91 | 66.5 | 37.9-95.1 | 90 | 1.5 | 1.4-1.6 | 90 | 28.2 | 16.6-39.8 |
|  | 2021 | Male | 76 | 34.4 | 1.8 67.1 | 74 | 72 | 48.4-95.7 | 74 | 1.6 | 1.5-1.8 | 74 | 27.0 | 18.8-35.2 |
|  | 2021 | Total | 168 | 34.5 | 2.8 66.3 | 165 | 69 | 42.0-96.0 | 164 | 1.6 | 1.4-1.7 | 164 | 27.6 | 17.4-37.9 |
| Parakanã | 2007 | Female | 71 | 35.2 | 7.8 62.7 | 66 | 52.3 | 37.3-67.3 | 66 | 1.5 | 1.4-1.6 | 66 | 23.5 | 17.3-29.8 |
|  | 2007 | Male | 58 | 39.7 | 8.7 70.6 | 57 | 60.7 | 46.6-74.9 | 57 | 1.6 | 1.5-1.7 | 57 | 23.6 | 18.2-29.1 |
|  | 2007 | Total | 129 | 37.2 | 8.0 66.5 | 123 | 56.2 | 39.5-72.9 | 123 | 1.5 | 1.4-1.7 | 123 | 23.6 | 17.7-29.4 |
|  | 2021 | Female | 75 | 39.5 | 7.5 71.5 | 67 | 61.5 | 36.6-86.4 | 46 | 1.5 | 1.3-1.7 | 47 | 26.7 | 17.5-36.0 |
|  | 2021 | Male | 87 | 35.8 | 5.9 65.6 | 75 | 63.9 | 30.9-96.9 | 45 | 1.5 | 1.4-1.7 | 38 | 24.7 | 16.7-32.7 |
|  | 2021 | Total | 162 | 37.5 | 6.5 68.5 | 142 | 62.8 | 33.3-92.2 | 91 | 1.5 | 1.4-1.7 | 85 | 25.8 | 16.9-34.7 |
| Gavião | 2007 | Female | 55 | 33 | 3.4 62.6 | 37 | 65.8 | 39.6-92.0 | 37 | 1.6 | 1.4-1.7 | 37 | 26.6 | 16.4-36.9 |
|  | 2007 | Male | 42 | 37.1 | 7.1 67.1 | 24 | 71.6 | 38.7-104.4 | 22 | 1.7 | 1.6-1.8 | 22 | 26.5 | 18.6-34.3 |
|  | 2007 | Total | 97 | 34.8 | 4.9 64.7 | 61 | 68.1 | 38.8-97.3 | 59 | 1.6 | 1.4-1.8 | 59 | 26.6 | 17.2-35.9 |
|  | 2021 | Female | 78 | 41 | 6.7 75.3 | 69 | 75.3 | 47.7-102.9 | 67 | 1.6 | 1.5-1.8 | 65 | 29 | 19.9-38.1 |
|  | 2021 | Male | 74 | 42.9 | 5.8 80.0 | 65 | 73.9 | 39.3-108.5 | 58 | 1.6 | 1.4-1.9 | 57 | 29.1 | 19.9-38.3 |
|  | 2021 | Total | 154 | 42 | 6.5 77.4 | 136 | 74.6 | 43.7-105.5 | 127 | 1.6 | 1.4-1.8 | 124 | 29.0 | 19.9-38.1 |
