# Supplementary material for: Fourteen-year trends in overweight, general obesity, and abdominal obesity in Amazonian indigenous peoples
Source: BMC Public Health. 2024 May 1;24:1210. doi: 10.1186/s12889-024-18689-2 (PMC11064236; doi:10.1186/s12889-024-18689-2)
Supplement: Supplementary file 2 — Supplementary Material 2. [file 12889_2024_18689_MOESM2_ESM.docx]

Table S2. Nutritional status profile by sex in adults of seven indigenous people from the Brazilian Amazon in 2007 and 2021.

| People | Year | Gender | Low weight | | | Overweight | | Obesity | | Central obesity | | |
| --- | --- | --- | --- | --- | --- | --- | --- | --- | --- | --- | --- | --- |
|  |  |  | N | % | IC95% | % | IC95% | % | IC95% | N | % | IC95% |
| Arara | 2007 | Female | 33 | 6.1 | -2.1 -14.2 | 21.2 | 7.3 -35.2 | 0.0 | 0.0 -0.0 | 24 | 50.0 | 30.0 -70.0 |
|  |  | Male | 32 | 0.0 | 0.0 -0.0 | 15.6 | 3.0 -28.2 | 3.1 | -2.9 -9.2 | 20 | 0.0 | 0.0 -0.0 |
|  |  | Total | 65 | 3.1 | -1.1 -7.3 | 18.5 | 9.0 -27.9 | 1.5 | -1.5 -4.5 | 64 | 18.5 | 9.0 -27.9 |
|  | 2021 | Female | 45 | 4.4 | -1.6 -10.5 | 40.0 | 25.7 -54.3 | 4.4 | -1.6 -10.5 | 0 | 0 | 0 -0 |
|  |  | Male | 37 | 0.0 | 0.0 -0.0 | 35.1 | 19.8 -50.5 | 2.7 | -2.5 -7.9 | 0 | 0 | 0 -0 |
|  |  | Total | 82 | 2.4 | -0.9 -5.8 | 37.8 | 27.3 -48.3 | 3.7 | -0.4 -7.7 | 0 | 0 | 0 -0 |
| Araweté | 2007 | Female | 30 | 6.7 | -2.3 -15.6 | 13.3 | 1.2 -25.5 | 0.0 | 0.0 -0.0 | 30 | 46.7 | 28.8 -64.5 |
|  |  | Male | 35 | 0.0 | 0.0 -0.0 | 2.9 | -2.7 -8.4 | 0.0 | 0.0 -0.0 | 34 | 0.0 | 0.0 -0.0 |
|  |  | Total | 65 | 3.1 | -1.1 -7.3 | 7.7 | 1.2 -14.2 | 0.0 | 0.0 -0.0 | 64 | 21.5 | 11.5 -31.5 |
|  | 2021 | Female | 113 | 13.3 | 7.0 -19.5 | 15.0 | 8.5 -21.6 | 2.7 | -0.3 -5.6 | 115 | 45.2 | 36.1 -54.3 |
|  |  | Male | 101 | 6.9 | 2.0 -11.9 | 16.8 | 9.5 -24.1 | 1.0 | -0.9 -2.9 | 100 | 8.0 | 2.7 -13.3 |
|  |  | Total | 214 | 10.3 | 6.2 -14.3 | 15.9 | 11.0 -20.8 | 1.9 | 0.1 -3.7 | 215 | 28.0 | 22.0 -34.1 |
| Asurini do Xingu | 2007 | Female | 22 | 4.5 | -4.2 -13.2 | 45.5 | 24.6 -66.3 | 27.3 | 8.7 -45.9 | 22 | 95.5 | 86.8 -104.2 |
|  |  | Male | 15 | 0.0 | 0.0 -0.0 | 53.3 | 28.1 -78.6 | 20.0 | -0.2 -40.2 | 15 | 53.3 | 28.1 -78.6 |
|  |  | Total | 37 | 2.7 | -2.5 -7.9 | 48.6 | 32.5 -64.8 | 24.3 | 10.5 -38.1 | 37 | 78.4 | 65.1 -91.6 |
|  | 2021 | Female | 29 | 3.4 | -3.2 -10.1 | 55.2 | 37.1 -73.3 | 13.8 | 1.2 -26.3 | 29 | 79.3 | 64.6 -94.1 |
|  |  | Male | 28 | 0.0 | 0.0 -0.0 | 82.1 | 68.0 -96.3 | 17.9 | 3.7 -32.0 | 29 | 86.2 | 73.7 -98.8 |
|  |  | Total | 57 | 1.8 | -1.7 -5.2 | 68.4 | 56.4 -80.5 | 15.8 | 6.3 -25.3 | 58 | 84.2 | 74.7 -93.7 |
| Kararaô | 2007 | Female | 7 | 0.0 | 0.0 -0.0 | 71.4 | 38.0 -104.9 | 14.3 | -11.6 -40.2 | 7 | 85.7 | 59.8 -111.6 |
|  |  | Male | 4 | 0.0 | 0.0 -0.0 | 75.0 | 32.6 -117.4 | 0.0 | 0.0 -0.0 | 4 | 50.0 | 1.0 -99.0 |
|  |  | Total | 11 | 0.0 | 0.0 -0.0 | 72.7 | 46.4 -99.0 | 9.1 | -7.9 -26.1 | 11 | 72.7 | 46.4 -99.0 |
|  | 2021 | Female | 16 | 0.0 | 0.0 -0.0 | 75.0 | 53.8 -96.2 | 31.3 | 8.5 -54.0 | 16 | 81.3 | 62.1 -100.4 |
|  |  | Male | 7 | 0.0 | 0.0 -0.0 | 85.7 | 59.8 -111.6 | 42.9 | 6.2 -79.5 | 7 | 85.7 | 59.8 -111.6 |
|  |  | Total | 23 | 0.0 | 0.0 -0.0 | 78.3 | 61.4 -95.1 | 34.8 | 15.3 -54.2 | 23 | 82.6 | 67.1 -98.1 |
| Xikrin do Bakajá | 2007 | Female | 80 | 0.0 | 0.0 -0.0 | 33.8 | 23.4 -44.1 | 8.8 | 2.6 -14.9 | 77 | 74.0 | 64.2 -83.8 |
|  |  | Male | 77 | 2.6 | -1.0 -6.2 | 35.1 | 24.4 -45.7 | 2.6 | -1.0 -6.2 | 78 | 25.6 | 16.0 -35.3 |
|  |  | Total | 157 | 1.3 | -0.5 -3.0 | 34.4 | 27.0 -41.8 | 5.7 | 2.1 -9.4 | 155 | 49.0 | 41.2 -56.9 |
|  | 2021 | Female | 90 | 3.3 | -0.4 -7.0 | 66.7 | 56.9 -76.4 | 37.8 | 27.8 -47.8 | 87 | 82.8 | 74.8 -90.7 |
|  |  | Male | 74 | 0.0 | 0.0 -0.0 | 67.6 | 56.9 -78.2 | 23.0 | 13.4 -32.6 | 70 | 54.3 | 42.6 -66.0 |
|  |  | Total | 164 | 1.8 | -0.2 -3.9 | 67.1 | 59.9 -74.3 | 31.1 | 24.0 -38.2 | 157 | 67.1 | 59.9 -74.3 |
| Parakanã | 2007 | Female | 66 | 4.5 | -0.5 -9.6 | 30.3 | 19.2 -41.4 | 3.0 | -1.1 -7.2 | 69 | 59.4 | 47.8 -71.0 |
|  |  | Male | 57 | 0.0 | 0.0 -0.0 | 15.8 | 6.3 -25.3 | 3.5 | -1.3 -8.3 | 58 | 8.6 | 1.4 -15.8 |
|  |  | Total | 123 | 2.4 | -0.3 -5.2 | 23.6 | 16.1 -31.1 | 3.3 | 0.1 -6.4 | 127 | 37.4 | 28.8 -45.9 |
|  | 2021 | Female | 42 | 2.4 | -2.2 -7.0 | 52.4 | 37.3 -67.5 | 14.3 | 3.7 -24.9 | 34 | 47.1 | 30.3 -63.8 |
|  |  | Male | 44 | 2.3 | -2.1 -6.7 | 45.5 | 30.7 -60.2 | 13.6 | 3.5 -23.8 | 39 | 15.4 | 4.1 -26.7 |
|  |  | Total | 86 | 2.3 | -0.9 -5.5 | 48.8 | 38.3 -59.4 | 14.0 | 6.6 -21.3 | 83 | 25.6 | 16.4 -34.8 |
| Gavião | 2007 | Female | 37 | 2.7 | -2.5 -7.9 | 51.4 | 35.2 -67.5 | 24.3 | 10.5 -38.1 | 32 | 100.0 | 100.0 -100.0 |
|  |  | Male | 22 | 0.0 | 0.0 -0.0 | 54.5 | 33.7 -75.4 | 22.7 | 5.2 -40.2 | 18 | 44.4 | 21.5 -67.4 |
|  |  | Total | 59 | 1.7 | -1.6 -5.0 | 52.5 | 39.8 -65.3 | 23.7 | 12.9 -34.6 | 50 | 67.8 | 55.9 -79.7 |
|  | 2021 | Female | 65 | 0.0 | 0.0 -0.0 | 83.1 | 74.0 -92.2 | 41.5 | 29.6 -53.5 | 54 | 61.1 | 48.1 -74.1 |
|  |  | Male | 57 | 0.0 | 0.0 -0.0 | 82.5 | 72.6 -92.3 | 42.1 | 29.3 -54.9 | 54 | 38.9 | 25.9 -51.9 |
|  |  | Total | 122 | 0.0 | 0.0 -0.0 | 82.3 | 75.5 -89.0 | 41.1 | 32.5 -49.8 | 118 | 43.5 | 34.8 -52.3 |
